# Multimodal Coherent Imaging of Retinal Biomarkers of Alzheimer’s Disease in a Mouse Model

**DOI:** 10.1038/s41598-020-64827-2

**Published:** 2020-05-13

**Authors:** Ge Song, Zachary A. Steelman, Stella Finkelstein, Ziyun Yang, Ludovic Martin, Kengyeh K. Chu, Sina Farsiu, Vadim Y. Arshavsky, Adam Wax

**Affiliations:** 1 Department of Biomedical Engineering, Duke University, Durham, NC 27708 USA; 2 Department of Ophthalmology, Duke Eye Center, Durham, NC 27710 USA

**Keywords:** Biomedical engineering, Imaging and sensing

## Abstract

We acquired depth-resolved light scattering measurements from the retinas of triple transgenic Alzheimer’s Disease (3xTg-AD) mice and wild type (WT) age-matched controls using co-registered angle-resolved low-coherence interferometry (a/LCI) and optical coherence tomography (OCT). Angle-resolved light scattering measurements were acquired from the nerve fiber layer, outer plexiform layer, and retinal pigmented epithelium using image guidance and segmented thicknesses provided by co-registered OCT B-scans. Analysis of the OCT images showed a statistically significant thinning of the nerve fiber layer in AD mouse retinas compared to WT controls. The a/LCI scattering measurements provided complementary information that distinguishes AD mice by quantitatively characterizing tissue heterogeneity. The AD mouse retinas demonstrated higher mean and variance in nerve fiber layer light scattering intensity compared to WT controls. Further, the difference in tissue heterogeneity was observed through short-range spatial correlations that show greater slopes at all layers of interest for AD mouse retinas compared to WT controls. A greater slope indicates a faster loss of spatial correlation, suggesting a loss of tissue self-similarity characteristic of heterogeneity consistent with AD pathology. Use of this combined modality introduces unique tissue texture characterization to complement development of future AD biomarker analysis.

## Introduction

There are currently 5.8 million Americans living with Alzheimer’s Disease (AD), with AD recently becoming the sixth largest cause of death in the US^1^. Estimated lifetime risk for AD increases significantly with age, with roughly 10% risk for men and 20% risk for women at the age of 65^2^. Neuronal and synaptic losses due to AD lead to cognitive impairment and dementia^3^, significantly affecting the quality of life of AD patients. With increasing life expectancy, the prevalence of AD is estimated to increase significantly worldwide, increasing the demand for comprehensive health care services^4^. Efforts to clinically diagnose AD by extracting relevant biomarkers can greatly improve the potential of an early and effective intervention^5^. Drug development efforts to minimize neuronal damage often recruit AD biomarker tests to achieve targeted effects^6^. Combination of biomarker tests for AD potentially allows for early intervention to improve cognitive function and slow the rate of cognitive decline^7^. A review of various physical activities for patients with dementia have shown significant improvement of cognitive function, up to 0.64 in standardized mean difference of cognitive test scores between intervention and control groups^8^. Current challenges however lie in the lack of quantifiable changes which predict AD early, aside from analysis of cognitive deficits, to allow for a clinical diagnosis^9,10^.

Definitive diagnosis of AD currently requires post-mortem analysis of brain sections^11^. The best-correlated pathological change in AD brains is the development of neurofibrillary tangles^12^, whose accummulation correlates with neuronal dysfunction and inflammation that eventually causes dementia^3^. Extracellular accumulation of amyloid-beta plaques is another strong correlate of AD^13–16^. While quantitative biomarkers have been observed with neuroimaging techniques such as MRI, SPECT and FDG-PET^5^, these methods remain costly and often invasive^17,18^. Many researchers have shown that the retina, which is an extension of the central nervous system, also develops visual anomalies from AD^19,20^. Studies from transgenic mouse models have drawn connections between retinal and brain pathology^21^. Specifically, plaque depositions in AD transgenic mouse retinas have been observed mainly in regions within the nerve fiber layer (NFL) and the outer plexiform layer (OPL)^22^. These observations point to a potential to monitor AD development through the pathological changes in the retina. Recent advancements in retinal imaging techniques, such as optical coherence tomography (OCT), allow potential noninvasive assessments of AD biomarkers^23–28^. Furthermore, the increasingly low-cost and accessible nature of retinal imaging gives rise to novel approaches in population-scale screening^29^. Efforts should therefore be made to extract AD biomarkers using retinal imaging to visualize changes associated with pathology.

In this study, we examined light scattering parameters of *ex vivo* transgenic mouse retinas using angle-resolved low-coherence interferometry (a/LCI) guided by OCT. OCT acquires depth-resolved cross-sectional images of the retina with micron-level resolution^30–32^. Previous longitudinal studies have used OCT measurements of the retina to associate decreased layer thicknesses to AD, suggesting its potential as a diagnostic biomarker^33,34^. a/LCI is an optical technique which detects depth-resolved angular scattering distributions from tissue, and has demonstrated high clinical diagnostic accuracy in Barrett’s esophagus^35^ and cervical dysplasia^36^. Although a/LCI is not itself an imaging modality, it can be combined with the image guidance of OCT to extract unique light scattering features from distinct layers of the retinal tissue^37^. Co-registration of OCT and a/LCI to an identical imaging volume yields complementary information, specifically by allowing localization of regions of interest in the retina, followed by a/LCI analysis of light scattering parameters. As shown in the literature, angular scattering data from a/LCI may be used to compute tissue spatial correlation function that is sensitive to spatial features as small as 100 nm^38^ and as large as 200 μm^39^.

Here we used this combined system to perform comparative retinal imaging of the “triple transgenic” mouse model of AD and age-matched wild type control mice aiming to extract measurable changes that have the potential to serve as AD biomarkers. Retinal layer segmentation using OCT B-scans was performed to analyze corresponding light scattering data at the retinal layers of interest. Retinal texture at selective layers was characterized using a short-range spatial correlation metric, and angular scattering information was extended to examine tissue scattering intensity distributions. We discuss the potential of using these a/LCI light scattering parameters to complement known morphological changes associated with AD. Use of the combined imaging system offers a unique analysis that is otherwise unavailable using a single modality, which provides a more holistic account of potential AD biomarkers.

## Methods

### Animals

Mouse care and experiments were performed in accordance with procedures approved by the Institutional Animal Care and Use Committee of Duke University. B6C3-Tg (APPswe, PSEN1dE9), 85Dbo/Mmjax mice (also known as APP/PS1^40^) were purchased from Jackson Labs (stock #004462). They are double transgenic mice expressing a chimeric mouse/human amyloid precursor protein (Mo/HuAPP695swe) and a mutant human presenilin 1 (PS1-dE9), both directed to CNS neurons. WT controls were littermates from heterozygous breeding, which did not carry APP and PS1 transgenes. We initially sought to utilize this mouse AD model, which is most commonly used in studies of the brain. However, morphometric examination of their retinas revealed a gross defect consisting of age-dependent formation of retinal folds (see Supplementary Fig. S1). This defect could not be attributed to the presence of AD-associated transgenes because it was also observed in their WT littermates (see Supplementary Fig. S1). The severity of retinal distortion was too significant for these mice to be utilized in the a/LCI analysis, and we were not able to effectively breed out the underlying mutation without knowing its nature. Therefore, we resorted to adopting an alternative “triple transgenic” (3xTg) mouse AD model. B6;129-Tg (APPSwe, tauP301L)1Lfa *Psen1*^*tm1Mpm*^/Mmjax mice described in^41,42^ (also known as 3xTg-AD, hereafter referred to as AD mice) were characterized by normal retinal morphology up to 15–16 months of age and were purchased from Jackson Labs (stock # 034830). Because it was previously reported that, at least in the brain, female mice of this line are more predisposed to forming amyloid deposits than males, only female mice were used in our experiments. B6129SF2/J mice were purchased from Jackson Labs (stock # 101045) and used as a control line. These control mice were F2 offspring from the F1 × F1 breeding between C57BL/6 J females (B6) and 129S1/SvImJ males. All mice were tested for the lack of Rd1 and Rd8 mutations.

### Sample preparation

Mouse retinal imaging was performed on freshly excised, intact eyeballs from age-matched WT and triple transgenic (3xTg-AD) mice. The triple transgenic mice were homozygous for all three mutant alleles (Psen1 mutation, co-injected APPSwe and tauP301L transgenes), which translate to traits that are thought to be defining features of AD^41^. The triple transgenic mice used in this study were beyond 15 months of age and well accepted for the study of AD pathology.

Mice were euthanized with carbon dioxide asphyxiation, followed by decapitation. Eyes were removed from the skull with the optic nerve intact and placed in cold Ringer’s solution. Before imaging, each eye was cleared of connective tissue and the superior quadrant was marked. The eye was then positioned with the superior quadrant aligned with the positive vertical scan axis in a custom-designed chamber and stabilized with grease^37^. The chamber design accounted for the eye curvature, which maintained its alignment relative to the custom objective during imaging. The chamber was placed on a translation stage that allows for X, Y and Z movements for alignment and pathlength matching as required for interferometry. Both the eye and the custom objective were immersed in Ringer’s solution during imaging.

### Histological techniques

Retinal morphology was evaluated in semi-thin plastic-embedded retinal cross-sections (0.5 μm thick) obtained from mice after a/LCI and OCT imaging. Sections were prepared as described in Lobanova *et al*.^43,44^ and stained with toluidine blue for light microscopy. For immunohistochemistry, agarose-embedded retinal cross-sections were prepared and collected in 24-well plates^45^, and incubated overnight with biotinylated anti-β-amyloid, primary antibody (6E10; Covance # SIG-39340), followed by 2 h incubation with DyLight streptavidin 488 secondary antibody (Vector laboratories #SA-5488) in PBS containing 0.1% Triton X-100. Sections were washed three times in PBS, mounted with Fluoromount G (Electron Microscopy Sciences) under glass coverslips, and visualized using a Nikon Eclipse 90i confocal microscope.

### Combined imaging system

The co-registered a/LCI and OCT system used in this study was described in a previous publication^37^. To summarize, horizontally polarized light from a Ti:Sapphire laser (Coherent Mira-900F, λ = 830 nm) is passed through a half-wave plate prior to coupling into a spool of polarization maintaining fiber (Corning, 50 m). Self-phase modulation within the fiber spectrally broadens the light to allow for improved depth resolution via low-coherence interferometry^46^. Spectrally broadened light is then split into the sample and reference arms of a Mach-Zehnder interferometer. In each arm, the polarization state of the light was optimized through geometric manipulation of the fiber using polarization-controlling paddles. The a/LCI beam in the sample arm is collimated and delivered to the sample at an oblique angle. Translation of the mirror and lens before BS1 allowed for precise control of the illumination angle. To perform retinal imaging, a custom objective (Wasatch Photonics) was inserted between the a/LCI objective and the crystalline lens of the eye. The custom objective was placed in contact with the front surface of the eyeball during imaging, forming a 4f-system with the eye, such that the a/LCI illumination is imaged to recreate a collimated beam at the retina. The objective takes into account the refraction from both the crystalline lens and the cornea of the eye under immersion conditions. Light scattered by the retina is propagated to BS2, where it is combined with the reference field before being detected by an imaging spectrometer (Princeton Instruments, SP-2150). A galvanometer placed in the scattered path scans the angle-resolved scattered field over a range of solid angles. The translation of the retro-reflector prism in the reference arm during retinal imaging allowed for pathlength matching to account for variance in optical pathlength among the population of eyes.

**Figure 1:**
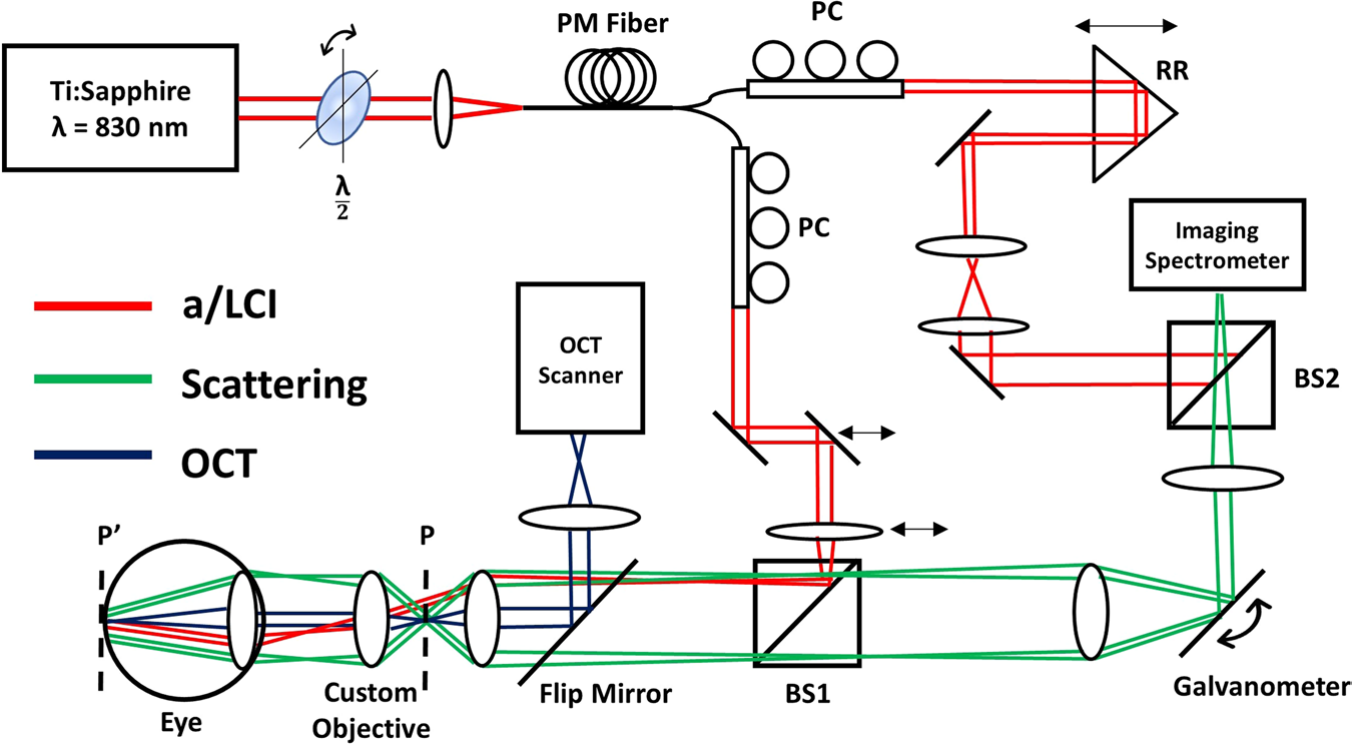
Schematic of combined a/LCI and OCT system for mouse retinal imaging. PM Fiber = Polarization Maintaining Fiber, PC = Polarization Control, RR = Retro Reflector, BS = Beamsplitter, P = Sample Plane, P’ = Sample Plane after insertion of the custom objective. Red lines show a/LCI illumination path, striking the retina with an oblique collimated beam. Green lines show angle-resolved scattered light from retina. Blue lines show OCT beam path for imaging of the retina.

Integration of an OCT system (Wasatch Photonics, Spark, λ = 830 nm, Δλ = 155 nm, A-line rate = 40 kHz) provided real time guidance during imaging. A flip mirror was introduced in the sample path to allow for sequential acquisitions of OCT B-scans and a/LCI measurements from the same tissue location. Registration of the a/LCI beam with the OCT field of view (FOV) was achieved by imaging a 1 mm diameter pinhole placed at the a/LCI sample plane. The 400 µm a/LCI beam was centered on the pinhole, and the pinhole was then centered on the OCT FOV at the same sample plane in depth corresponding to a/LCI, where the scattering signal was maximized. A schematic of the co-registered system for mouse retinal imaging is shown in Fig. 1.

### Data acquisition and processing

**Figure 2:**
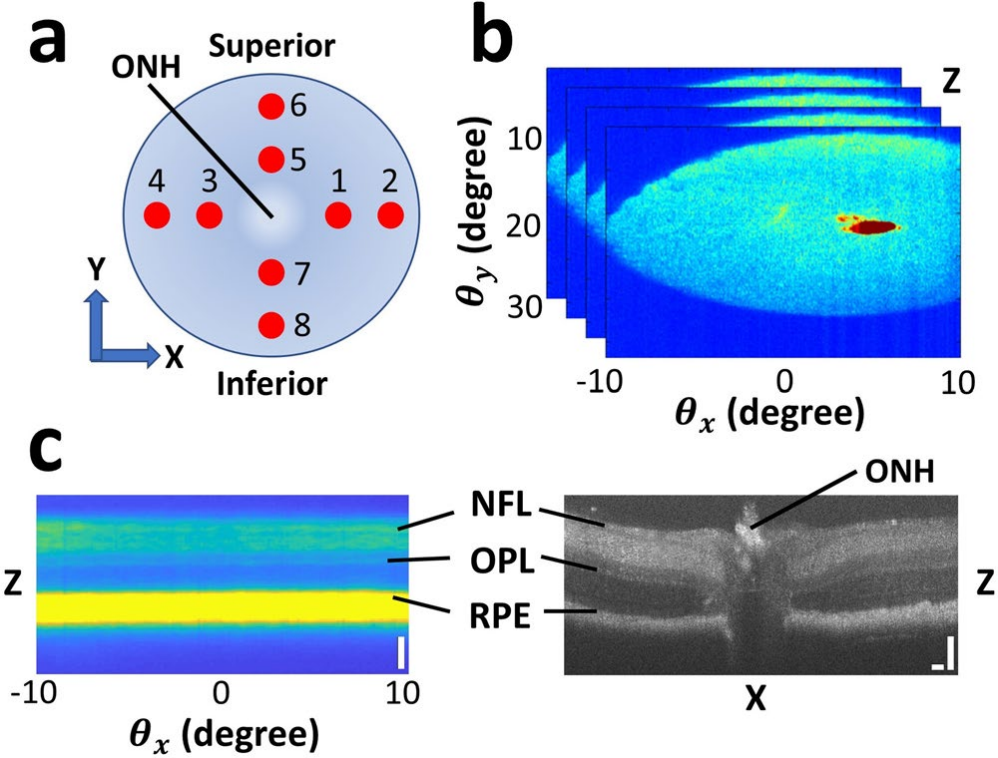
Schematic of data acquisition. (**a**) Locations on the retina on which a/LCI and OCT scans were acquired. ONH marks the optic nerve head. The eye is positioned with the superior quadrant aligned with the +Y axis. At each location, a single a/LCI scan and a vertical and horizontal OCT B-scan were performed. (**b**) Example of 2-dimensional angular scattering acquired using a/LCI with coherence-gated depth for one scan position. The small “red spot” is an artifact of specular reflection, which was manually segmented and excluded in Fig. 2c and subsequent analysis. (**c**) Side-by-side comparison of an OCT B-scan and a depth vs. angle a/LCI measurement corresponding to one location on the retina. NFL = nerve fiber layer; OPL = outer plexiform layer, RPE = retinal pigmented epithelium. Scale bars are 100 µm.

For retinal imaging, OCT imaging was first used to align the eyeball by placing the optic nerve head (ONH) at the center of the FOV in both horizontal and vertical scan directions. Using the ONH as a reference point, the retina can be sampled with the a/LCI beam at various locations of interest. Because the OCT and a/LCI beams are registered at the same image plane in depth (Z), translation of the eyeball and the custom objective in X and Y allowed for pinpointing of the locations of interest during data acquisition. Drying and deterioration of the eye after removal imposed a temporal window of ~20 minutes, within which all images must be acquired. To balance comprehensive imaging with sample quality, 8 locations were sampled on the retina using the combined system. Figure 2a illustrates the locations of interest at which data acquisition was performed. The superior quadrant of the eye was aligned with the +Y scan axis of OCT. The locations of interest were then sampled at 500 µm intervals along the horizontal and vertical axes. At each location, a single a/LCI measurement and a horizontal and vertical OCT B-scan were performed. The acquisition time for each 1D a/LCI scattering measurement is 4 ms and the entire 2-dimensional field is acquired over 600 angular scans in approximately 2.4 seconds. Total experiment time to image one eye amounted to 10–15 mins, which included OCT image acquisition, sample translation across the 8 locations, and corresponding a/LCI measurements.

Figure 2b illustrates a representative a/LCI measurement at one location of interest on the retina, showing 2-dimensional angular scattering for a specific range in depth. ${{\rm{\theta }}}_{{\rm{x}}}$ represents scattering along X, the dimension along which each 1D scattering measurement is made. The galvanometer scans the imaged scattering distribution along the range of ${{\rm{\theta }}}_{{\rm{y}}}$ to construct the 2D distribution. An artifact of specular reflection from the crystalline lens of the eye appears as a small “red spot” in Fig. 2b, which was manually segmented and excluded from subsequent data analysis. Each 1D a/LCI scan gives the scattered intensity as a function of wavelength and angle, which is transformed via simple Fourier transform along the spectral dimension to angle-resolved measurements of the scattered light as a function of depth. Consequently, 2-dimensional angle-resolved scattering from retinal structures may be measured with a coherence-gated depth sensitivity of several microns. This depth resolution allows for extraction of light scattering from specific retinal layers, which are easily identified using OCT. As shown in Fig. 2c, the NFL, OPL, and the retinal pigmented epithelium (RPE) can be precisely identified in a depth vs. ${{\rm{\theta }}}_{{\rm{x}}}$ scan using the corresponding layers in an OCT B-scan. The segmentation of the NFL, OPL, and RPE in the OCT images were accomplished manually using definition of retinal layer boundaries described in Srinivasan *et al*.^47^. Following OCT layer segmentation, the measured thicknesses can be used to identify the same layers in the light scattering data by scaling them with the a/LCI spectrometer pixel-to-depth conversion factor. Therefore, OCT retinal layer segmentation allowed for corresponding 2-dimensional angular scattering to be extracted from an a/LCI scan with high specificity.

**Figure 3:**
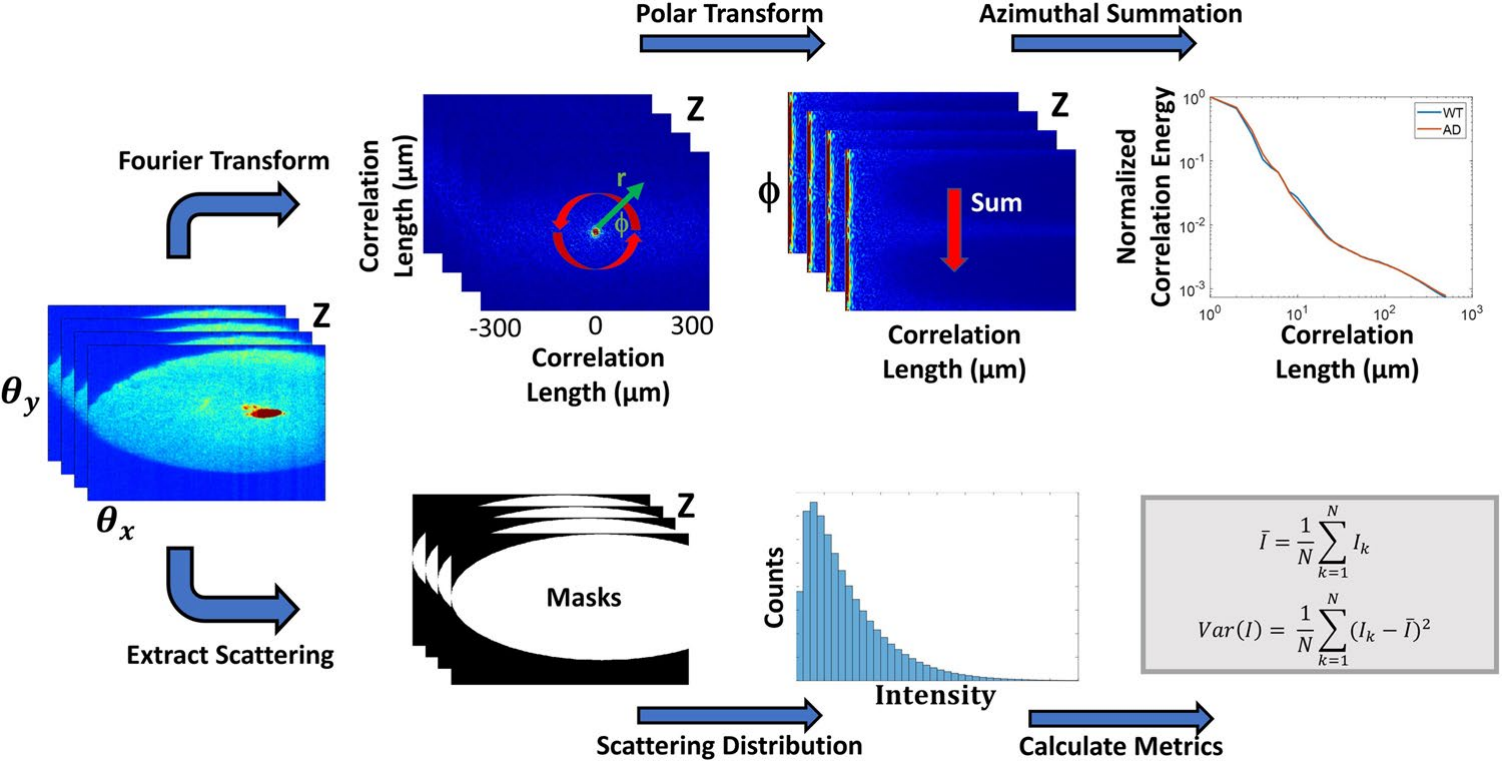
a/LCI data processing steps. For each depth-resolved angular scattering plane, two parallel processing pathways were taken to extract analytical metrics. Spatial correlation analysis is performed through a Fourier transform of the 2D angular scattering plane, allowing azimuthal summation and extraction of short- and long-range correlation slopes. Simultaneously, histogram analysis is performed by isolating the scattering distribution using binary masks and calculating statistical metrics. Equations for mean and variance of scattering intensity use k to denote pixel count and N for the total number of pixels captured within the 2-dimensional scattering distribution.

Tissue heterogeneity as a result of morphological and optical property changes will produce changes in light scattering. Using the 2D angular scattering data segmented from specific retinal layers, various analytical metrics may be computed. A summary of these processing steps is shown in Fig. 3. Previously, it was shown that the Fourier transform of the angular scattering distribution yields the two-point spatial correlation of the optical field^48^. Spatial correlation analysis reveals structure by describing tissue organization and homogeneity at various length scales. For anisotropic tissues where no directional information is evident, the 2D spatial correlation can be azimuthally integrated to extract radial correlation energy as a function of correlation lengths up to a few hundred microns. The correlation energy plot can be visualized in log-log scale at various retinal layers to draw comparisons between the WT and AD mouse retinas. Correlation energy at each length scale quantifies the relative contribution of that length scale to light scattering compared to the overall tissue structure. Specifically, the slope of the log-log curve can be related to a power-law exponent that describes a monotonically decreasing correlation of the tissue at increasing length scales. This power-law relationship has been used in a previous formalism for tissue fractal dimension^49^. The slopes of the correlation curve, termed α, were extracted at length scales corresponding to cellular and sub-cellular features (~2–10 µm) for both the AD and WT retinas at each histological layer. The differences in the slopes describe the rate of loss of spatial correlation of the retina at those length scales, shedding light on the loss of tissue homogeneity and texture.

Analysis of statistical properties of the angular scattering intensity can also characterize changes in tissue structure. Specifically, statistical parameters are generated from the histograms of the scattering signal from each retinal layer. A histogram of pixel intensities can be generated from the entire angular range of the 2-dimensional scattering acquired from each depth plane. The mean and variance of the angular scattering intensities were then calculated for the WT and AD mouse retinas for the NFL, OPL and RPE layers, and compared between WT and AD models. This process is shown in Fig. 3.

### Statistical analysis

We utilized mixed repeated measures analysis of variance to examine differences between scattering-based parameters and OCT layer thicknesses in the AD and WT populations. Various metrics extracted from OCT and a/LCI measurements are used to identify biophysical changes in AD mouse retinas compared to those in WT controls. Therefore, the test is appropriate to compare means between the two groups, while accounting for the correlation within the scans taken at the 8 different locations on each retina. Normality in the data was verified using the Shapiro-Wilk test (α = 0.003, Bonferroni correction applied for multiple comparisons). Homogeneity of error variances between the AD and WT groups was verified using the Levene’s test (α = 0.05). Visualization of the covariance matrix revealed a compound symmetric structure with a small correlation value (ρ = −0.1429) within the 8 sampled positions on each eye, indicating a slight degree of within-subjects dependence. For our analysis, 8 a/LCI scans taken from 10 WT retinas and 13 AD retinas were used, and 309 OCT B-scans were used for layer segmentation. 59 OCT B-scans were removed from statistical analysis due to insufficient contrast or inability to be properly segmented. Statistical significance for all tests uses α = 0.05. All statistical analysis was performed using JMP Pro (SAS, Cary, NC).

## Results

**Figure 4:**
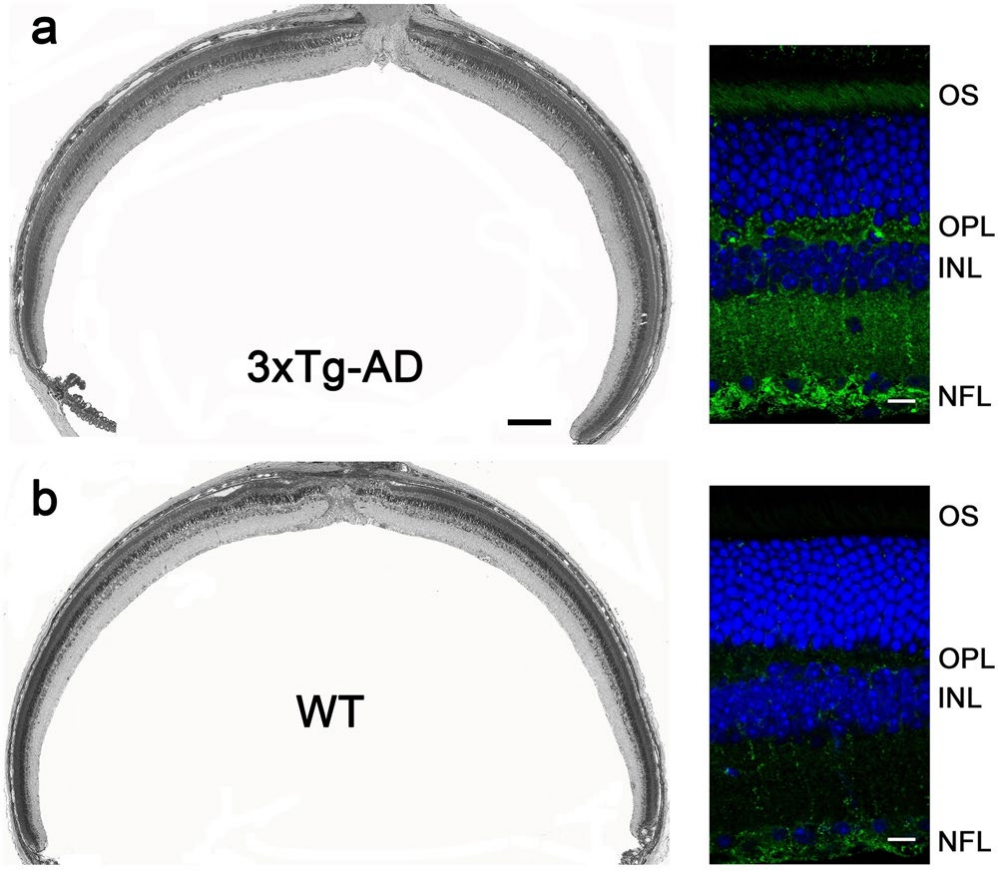
Morphometric analysis and amyloid immunostaining in the retinas of 15 months old 3xTg-AD (**a**) and WT (**b**) mice of the matched genetic background. Images on the left are obtained from 0.5 μm-thick plastic-embedded retinal cross-sections; scale bar: 200 μm. Images on the right are obtained from retinal agarose sections immunostained with anti-amyloid antibody 6E10 (green). Nuclei are stained with Hoechst (blue); scale bar: 10 μm.

Figure 4 presents morphologic and amyloid immunostaining images of 15 months old 3xTg-AD and age-matched WT retinas. As previously reported^42^, we observed amyloid deposits in the inner retina, located primarily in the OPL and NFL (Fig. 4a).

**Figure 5:**
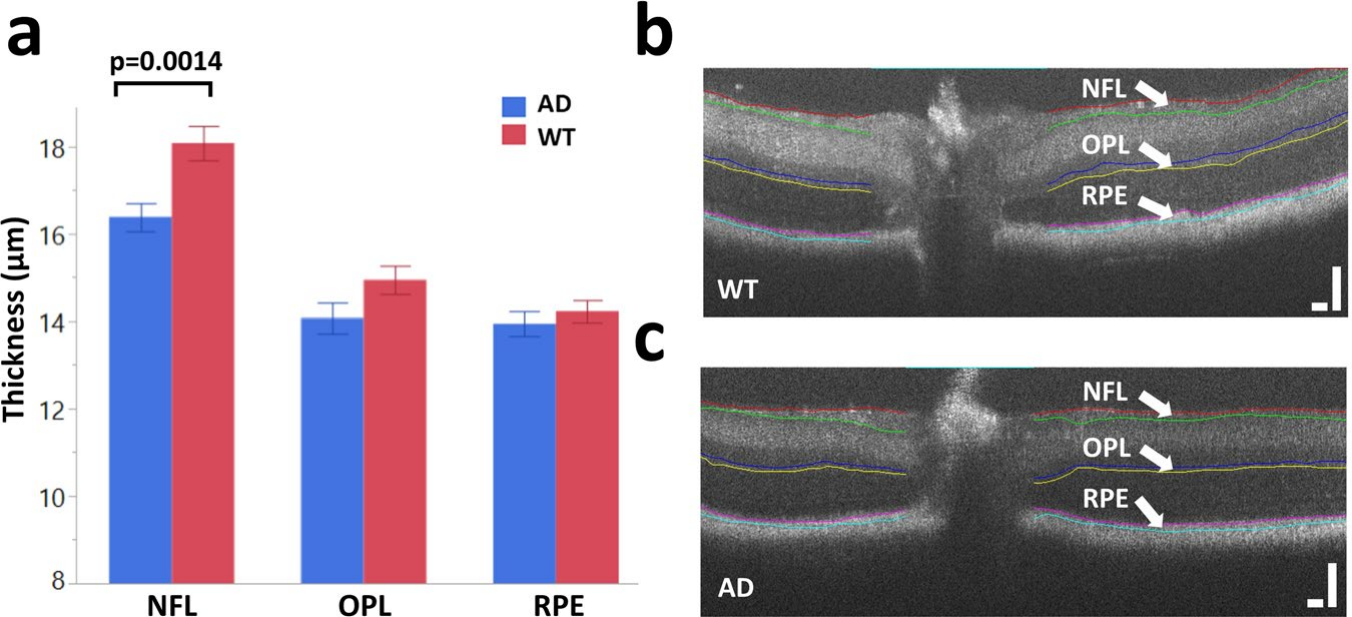
OCT retinal layer segmentation. (**a**) Average layer thicknesses of NFL, OPL and RPE for both wild type and AD mouse retinas post 15–16 months of age. Layer thickness measurements reveal a statistically significant thinning of the NFL for AD mouse retinas. Bars represent mean ± standard error. (**b**) Representative segmented OCT image of a wild type mouse retina. (**c**) Representative segmented OCT image of an AD mouse retina. Scale bars: 100 µm.

Figure 5 presents the segmentation results from our OCT B-scans of the AD mice and age-matched WT controls. Retinal layer locations extracted from OCT provide equivalent segmentation in the corresponding a/LCI measurements, which allows for well-matched layer specificity between the two modalities. For this experiment, NFL, OPL and RPE were chosen for analysis, as these three layers showed the greatest scattered signal intensity in the a/LCI measurements. The NFL is of particular interest, as mentioned earlier. The average layer thickness for NFL is significantly lower for AD mouse retinas (p = 0.0014) as seen in Fig. 5a. NFL thinning in AD models is consistent with results seen in the literature^50–52^. Analysis of variance revealed no significant quadrant-specific contributions to AD NFL thickness (p = 0.5936). Representative segmented OCT images of equal-aged WT mouse retina and of AD mouse retina are shown in Fig. 5b,c, respectively.

**Figure 6:**
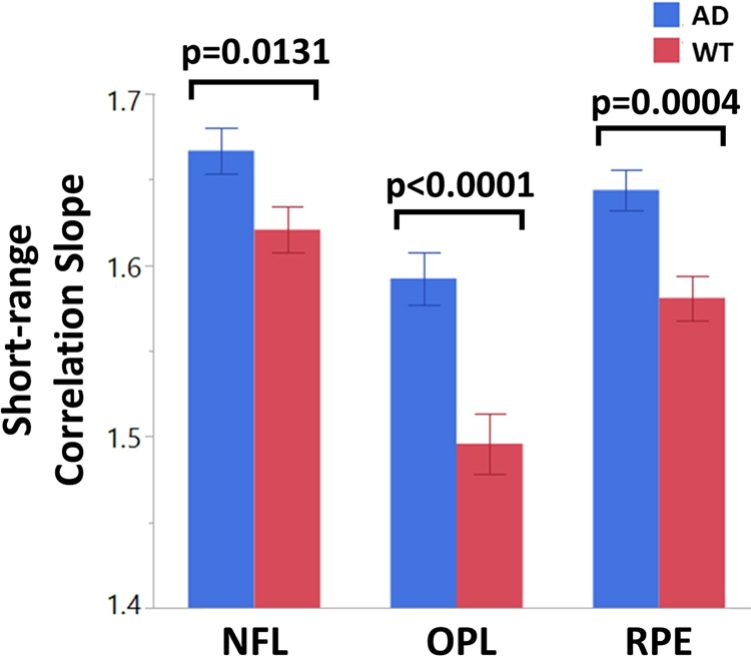
Short range correlation slopes for wild type and AD mouse retinas at various layers. AD mouse retinas exhibit a statistically higher correlation slope value across all layers, indicating a faster loss of spatial correlation. Bars represent mean ± standard error.

OCT segmentation results allow for subsequent light scattering analysis to be conducted at the respective retinal layers. 1-D correlation as a function of correlation length was extracted for each of the three layers. Figure 6 shows the average short-range correlation slope values between 2–10 µm for NFL, OPL, and RPE for both the AD and WT mouse retinas. Statistically significant differences were observed for all three retinal layers. Specifically, AD mouse retinas exhibit significantly higher slope values at short-range correlations, with p = 0.0131 for NFL, p < 0.0001 for OPL, and p = 0.0004 for RPE. The correlation slope is related to the tissue fractal dimension and is a direct indication of the rate of the loss of spatial correlation at various length scales. This model treats the retina as a continuous random medium that has reduced self-similarity at increasing length scales^53–57^. The higher slope seen for AD mouse retinas across all layers indicate a significantly faster loss of spatial correlation compared to WT retinas. Tissue heterogeneity therefore contributes to a reduction in spatial coherence that is observed in the AD retinal model.

**Figure 7:**
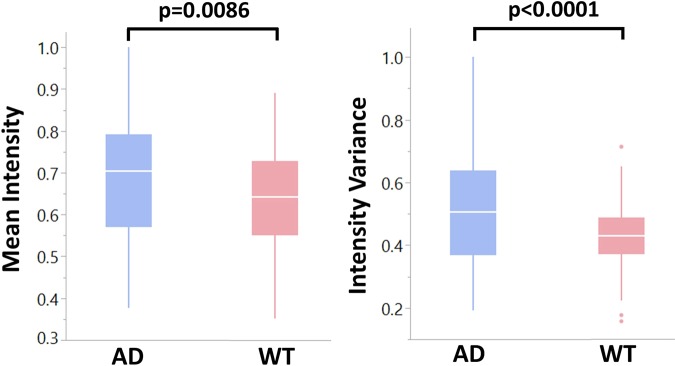
Normalized mean and variance of angular scattering intensity in the NFL for both AD and WT mouse retinas. The NFL in AD mouse retinas reveal a higher mean and variance in scattering intensity, resulting from tissue heterogeneity.

Histogram analysis of light scattering intensity was performed on the depth-resolved 2D scattering collected for each retinal layer. The mean and variance of the angular scattering intensity measured using a/LCI can be compared between the WT and AD mouse groups. The mean and variance of the light scattering distribution were calculated as described in Fig. 3 for all three retinal layers of interest. Statistical differences in scattering intensity between AD and WT retinas were not observed for the OPL and the RPE, but were observed for the NFL, consistent with previous studies showing more marked changes in this layer^58,59^. Figure 7 summarizes the differences in the NFL angular scattering for both AD and WT mouse retinas. Specifically, the NFL in AD mouse retinas exhibit a higher mean and variance in scattering intensity (p = 0.0086 for mean and p < 0.0001 for variance) compared to those in WT retinas.

## Discussion

Changes in retinal layer thicknesses from AD human patients have been observed in various studies using OCT^51,52^, suggesting the potential for a noninvasive approach to identify measurable changes that will aid in clinical diagnostics. The retina is generally more accessible for imaging than the brain, and retinal changes associated with AD encourage the advancement of optical screening technologies. The thinning of NFL in particular has been documented using OCT in many studies for AD patients in comparison to age-matched healthy controls^50–52^. Several studies have noted reduced mean NFL thickness, specifically in the superior quadrant, while others have reported a total retinal thickness reduction in AD^50,51^. Further, progress in the analysis of retinal vasculature organization has also been reported^60^. These morphological and visual defects are often associated with normal ageing and are not specific to AD, so we aimed to identify other retinal tissue parameters that have been altered specifically by AD pathology. However, instrumental variability resulting in contrast differences, as well as variability in segmentation protocols can make it difficult to compare OCT layer thickness, and thus biomarkers from other sources such as a/LCI could potentially improve diagnostic power.

The multimodal system used here allowed for analysis of depth-dependent angular light scattering, which can be used to evaluate structural information such as tissue spatial correlations. Image guidance from OCT enabled us to link a/LCI light scattering measurements to specific retinal layer depths. Further, a measurement scheme was established to consistently measure regions of interest by quadrant and distance from the optic nerve to enable comparison of coherent imaging measurements of the retina across a given animal population. To extract quantifiable changes in the retina with light scattering, we processed the angular scattering distributions to reveal tissue-specific descriptors. The combination of methods in the multimodal system along with the proposed measurement and analysis approach may provide unique AD biomarkers which can complement previously established parameters.

The a/LCI data is transformed into a two-point correlation function which is analyzed using a power law function. Specifically, an inverse power law behavior is indicative of tissue self-similarity which is quantified via fractal dimension (FD)^48^. The power law exponent, which is the slope of the integrated correlation function, is directly related to the fractal dimension by FD = 3 – α, where α is the slope^48^. This formalism treats the retina as a continuous structured medium that is perturbed by the AD pathology. Heterogeneity introduced by the pathology to the medium, therefore, disrupts its structure and organization. Analysis of the mouse retina using this method reveals significant differences in tissue correlation across all three layers of interest, in the NFL, OPL and RPE. Specifically, a greater average slope is observed for all three layers in the AD mouse retinas compared to the WT controls. A higher slope, or power law exponent, indicates a reduction in tissue fractal dimension. Physically, this signifies a faster loss in spatial correlation for AD mouse retinas at short-range length scales of 2–10 μm, corresponding to structures such as cells, organelles, and potentially to AD-related plaque depositions. Therefore, the measurement of the power law exponent can be used to quantify the degradation of tissue spatial structure resulting from pathology.

Angular scattering data from the retina can also be analyzed by extracting intensity distribution metrics. The findings presented here indicate an increased mean and variance in NFL scattering intensity for AD mouse retinas compared to the WT controls. We also examined the shapes of the scattering distribution histograms through skewness and kurtosis metrics, but were unable to find significant differences for any of the retinal layers. Increased scattering intensity is typically caused by an increase in the heterogeneity of the tissue’s refractive index, suggesting a change in structure. Previous studies using OCT to image diseased retinas in diabetic retinopathy, for example, have shown strong back reflections with high signal variance as a result of pathology^61^. A comparison may be drawn to the higher and more variable angular scattering in our AD mouse retinas, which may result from tissue heterogeneity as indicated from our earlier analysis of the power law exponent. Furthermore, variations in scattering intensity may also be attributed to the morphological change of layer thinning. For example, the average WT NFL thickness was roughly 18 µm, while the average AD NFL thickness was reduced to 16 µm. Because the axial resolution for light scattering measurements was roughly 10 µm, the axial point-spread-function (PSF) at the NFL boundary may push farther into the layer as it thins, presenting as an increased scattering within the layer. Further study is needed to link trends in light scattering intensity with layer thinning, but analysis of angular scattering distributions is nevertheless useful as a descriptor of tissue structural changes.

**Figure 8:**
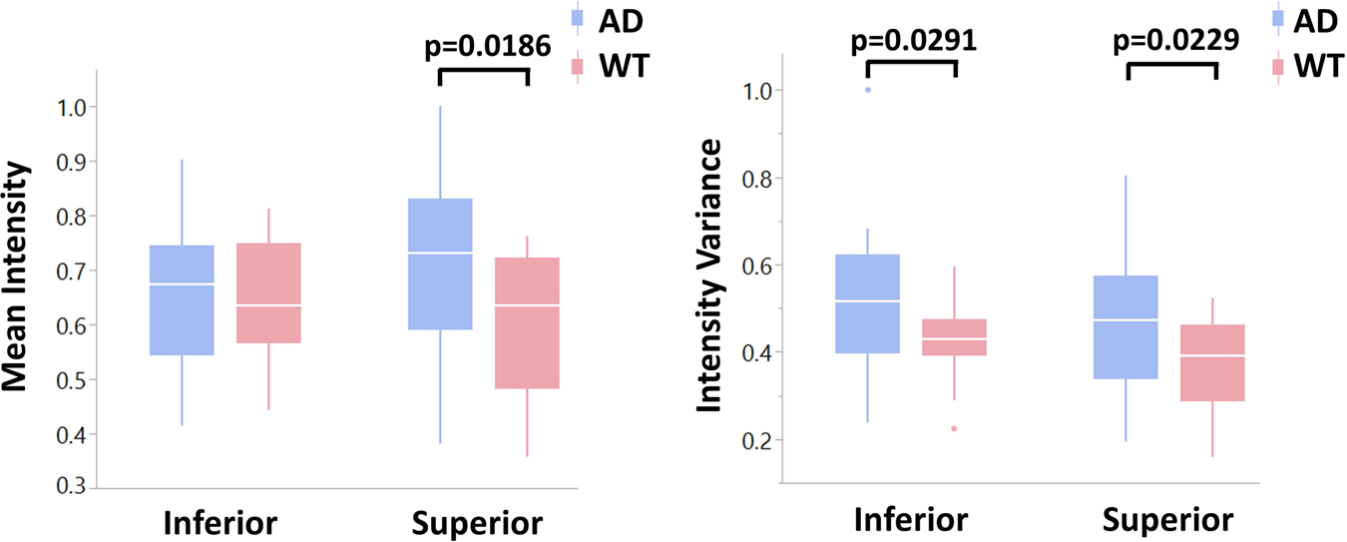
Normalized mean and variance of angular scattering intensity in the NFL for both AD and WT mouse retinas separated by quadrants. Differences in mean scattering intensity is largely attributed to the superior quadrant while variance has no quadrant-specific contributions.

Interestingly, our findings exhibit some quadrant specificity. In particular, analysis of scattering intensity from the NFL reveals quadrant-specific contributions. Figure 8 compares scattering intensity and variance between the inferior and superior quadrants of the NFL. It appears that differences in mean scattering intensity between AD and WT retinas are largely attributable to the superior quadrant, as significance was not observed in the inferior quadrant. This result complements several previous studies that link morphological changes in AD mouse models predominantly to the superior quadrant^52,62–64^. Variance in scattering intensity appears to show no quadrant-specific contributions, as both inferior and superior quadrants reveal significant differences.

A minor limitation of this work lies in the time constraint given by the post-mortem changes of the *ex vivo* eyeball, which limited the number of locations at which scanning was performed. Although the eye is kept in Ringer’s solution at proper temperatures following its removal from the skull, we found that our B-scan retinal layer contrast was substantially reduced after 15–20 minutes post-removal. This significantly reduces OCT image quality and prevents proper guidance for the subsequent a/LCI scan. Adequate sampling of the retina at 8 unique positions of interest requires quick but careful translation of the custom objective and the eye as a unit with high precision. Ideally, more locations would be sampled, including those at greater distances from the ONH within each quadrant of the retina. This could potentially enable further quadrant-specific comparisons to be made. Future work will focus on a spatial scanning mechanism that will be integrated with the co-registered system to enable sampling of the retina without physical translation of the eye. Use of optical scanning rather than mechanical methods will better facilitate rapid imaging and maximize the number of positions that may be sampled within the allotted time. Such an advancement is needed for potential *in vivo* measurements, which is currently also limited by the use of the flip mirror that introduces OCT imaging. The need of the flip mirror restricts the use of the two modalities in sequential mode, severely limiting faster scanning.

To conclude, we have utilized a multimodal imaging system to characterize changes in the optical properties of retina from the triple transgenic AD mouse model using a co-registered OCT and a/LCI system. The combined modalities allowed for extraction of unique measurable changes that are associated with the AD retinal model, pointing the way towards a potential biomarker. Using image guidance and subsequent layer segmentation from OCT, retinal layers can be reliably demarcated for further analysis using light scattering data obtained via a/LCI. Parameters were compared for light scattering distributions in AD and WT mouse retinas, and significant differences were found in mean and variance of scattering intensity, as well as short-range correlation. The layer-specific differences between pathological and control retinas are consistent with the known morphological changes associated with AD pathology. The data trends revealed in this study have a potential to serve as biomarkers of AD and, with further development, may enable a better identification of AD pathology in human patients.

## Supplementary information

Supplementary information.

## Data Availability

Images and datasets generated in this study are available from the corresponding author on reasonable request.
