# Supplementary material for: Multimodal Coherent Imaging of Retinal Biomarkers of Alzheimer’s Disease in a Mouse Model
Source: Sci Rep. 2020 May 13;10:7912. doi: 10.1038/s41598-020-64827-2 (PMC7220911; doi:10.1038/s41598-020-64827-2)

Multimodal Coherent Imaging of Retinal Biomarkers of Alzheimer's Disease in a Mouse Model

Ge Song[1*], Zachary A. Steelman[1], Stella Finkelstein[2], Ziyun Yang[1], Ludovic Martin[2], Kengyeh K. Chu[1], Sina Farsiu[1,2], Vadim Y. Arshavsky[2], Adam Wax[1]

[1]Department of Biomedical Engineering, Duke University, Durham, NC 27708, USA
[2]Department of Ophthalmology, Duke Eye Center, Durham, NC 27710, USA
*gs172@duke.edu

Supplementary Information

Supplementary Figure S1. Morphometric analysis of the retinas from (a) 14 months old APP/PS1 and (b) WT mice of matched genetic backgrounds. Images are obtained from 0.5 μm-thick plastic-embedded retinal cross-sections; scale bar: 200 μm.

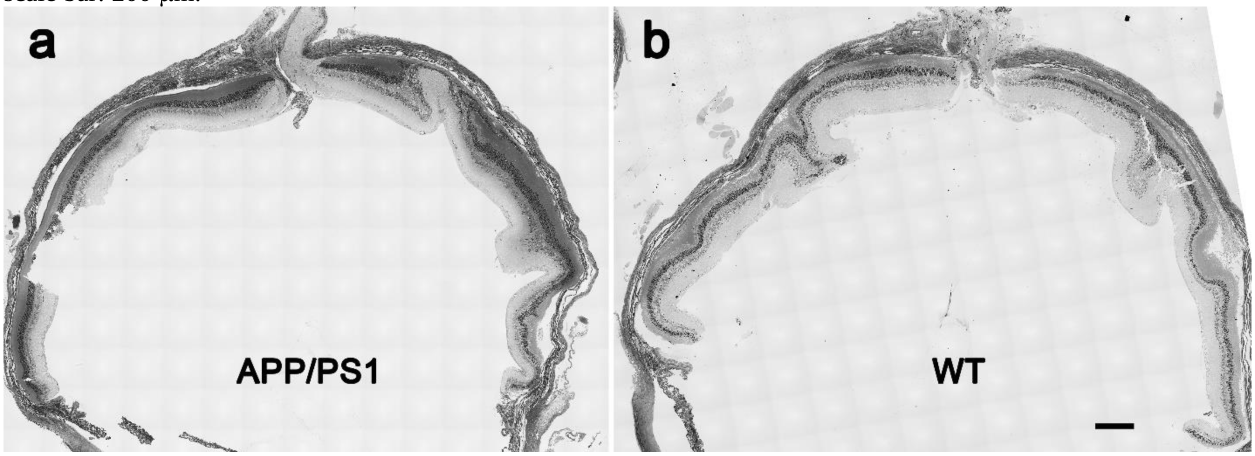

Supplement: Supplementary file 1 — Supplementary information. [file 41598_2020_64827_MOESM1_ESM.pdf]
